# STA9090 as a Potential Therapeutic Agent for Liver Fibrosis by Modulating the HSP90/TβRII/Proteasome Interplay: Novel Insights from In Vitro and In Vivo Investigations

**DOI:** 10.3390/ph16081080

**Published:** 2023-07-28

**Authors:** Osama A. Mohammed, Mustafa Ahmed Abdel-Reheim, Mohannad Mohammad S. Alamri, Jaber Alfaifi, Masoud I. E. Adam, Lobna A. Saleh, Alshaimaa A. Farrag, Amar Ibrahim Omer Yahia, Sameh Abdel-Ghany, AbdulElah Al Jarallah AlQahtani, Emad Bahashwan, Hanan B. Eltahir, Nahid A. Mohammed, Hend S. El-wakeel, Sara H. Hazem, Sameh Saber

**Affiliations:** 1 Department of Clinical Pharmacology, College of Medicine, University of Bisha, Bisha 61922, Saudi Arabia; 2 Department of Pharmaceutical Sciences, College of Pharmacy, Shaqra University, Aldawadmi 11961, Saudi Arabia; 3 Department of Pharmacology and Toxicology, Faculty of Pharmacy, Beni-Suef University, Beni Suef 62521, Egypt; 4 Department of Family Medicine, College of Medicine, University of Bisha, Bisha 61922, Saudi Arabia; malamri@ub.edu.sa; 5 Department of Child Health, College of Medicine, University of Bisha, Bisha 61922, Saudi Arabia; jalfaifi@ub.edu.sa; 6 Department of Medical Education and Internal Medicine, College of Medicine, University of Bisha, Bisha 61922, Saudi Arabia; mieadam@ub.edu.sa; 7 Department of Clinical Pharmacology, Faculty of Medicine, Ain Shams University, Cairo 11566, Egypt; lasaleh@tu.edu.sa; 8 Department of Pharmacology and Toxicology, Collage of Pharmacy, Taif University, Taif 21944, Saudi Arabia; 9 Department of Histology and Cell Biology, Faculty of Medicine, Assiut University, Assiut 71515, Egypt; alshaima@aun.edu.eg; 10 Unit of Anatomy, Department of Basic Medical Sciences, College of Medicine, University of Bisha, Bisha 61922, Saudi Arabia; 11 Unit of Pathology, Department of Basic Medical Sciences, College of Medicine, University of Bisha, Bisha 61922, Saudi Arabia; aiyahia@ub.edu.sa; 12 Department of Pathology, Faculty of Medicine and Health Sciences, University of Kordofan, Elobeid 11115, Sudan; 13 Department of Clinical Pharmacology, Faculty of Medicine, Mansoura University, Mansoura 35516, Egypt; samghany@mans.edu.eg; 14 Department of Internal Medicine, Division of Dermatology, College of medicine, University of Bisha, Bisha 61922, Saudi Arabia; aaljarallah@ub.edu.sa (A.A.J.A.); bahashwan@ub.edu.sa (E.B.); 15 Department of Basic Medical Sciences, College of Medicine, University of Bisha, Bisha 61922, Saudi Arabia; hbabiker@ub.edu.sa (H.B.E.); nahid@ub.edu.sa (N.A.M.); 16 Department of Biochemistry, Faculty of Medicine, University of El Imam, El Mahdi 11588, Sudan; 17 Department of Physiology, Faculty of Medicine, University of Gezira, Wad Madani 12217, Sudan; 18 Physiology Department, Benha Faculty of Medicine, Benha University, Qalubyia 13511, Egypt; h.elsayed@bu.edu.sa; 19 Physiology Department, Albaha Faculty of Medicine, Albaha University, Al-Baha 65779, Saudi Arabia; 20 Department of Pharmacology and Toxicology, Faculty of Pharmacy, Mansoura University, Mansoura 35516, Egypt; saraheshamhazem@gmail.com; 21 Department of Pharmacology, Faculty of Pharmacy, Delta University for Science and Technology, Gamasa 11152, Egypt

**Keywords:** liver fibrosis, STA9090, HSP90, TGF-β, TβRII, proteasome

## Abstract

Liver fibrosis is a progressive condition characterized by the build-up of fibrous tissue resulting from long-term liver injury. Although there have been advancements in research and treatment, there is still a need for effective antifibrotic medication. HSP90 plays a crucial role in the development of fibrosis. It acts as a molecular chaperone that assists in the proper folding and stability of TβRII, potentially regulating the signaling of TGF-β1. It has been established that TβRII can be degraded through the proteasome degradation system, either via ubiquitination-dependent or -independent pathways. In the present study, STA9090 demonstrated promising effects in both in vitro and in vivo models. It reduced LDH leakage, prolonged the survival rate of hepatocytes in rats with liver fibrosis, and improved liver function. Importantly, STA9090 exerted pleiotropic effects by targeting proteins involved in limiting collagen production, which resulted in improved microscopic features of the rat livers. Our findings suggest that STA9090-induced inhibition of HSP90 leads to the degradation of TβRII, a fibrogenic client protein of HSP90, through the activation of the 20S proteasomal degradation system. We also revealed that this degradation mechanism is not dependent on the autophagy–lysosomal pathway. Additionally, STA9090 was found to destabilize HIF-1α and facilitate its degradation, leading to the reduced transcription of VEGF. Moreover, STA9090’s ability to deactivate the NFκB signaling pathway highlights its potential as an anti-inflammatory and antifibrotic agent. However, further research is necessary to fully elucidate the underlying mechanisms and fully capitalize on the therapeutic benefits of targeting HSP90 and associated pathways.

## 1. Introduction 

Liver fibrosis is a consequence of long-term liver disease, like hepatitis, and can eventually lead to liver cirrhosis [1]. Despite advancements in diagnosing, managing, and treating liver fibrosis, the global prevalence of liver disease remains substantial in 2023, resulting in approximately 2 million deaths each year and accounting for 4% of all mortality [2]. Therefore, there is an ongoing requirement for efficacious antifibrotic medication and a deeper comprehension of the cellular and molecular processes involved in the regression of fibrosis.

Liver fibrosis is characterized by the accumulation of an excessive and abnormal extracellular matrix (ECM) [3]. The ECM, which consists of an intricate framework of proteins and other substances, plays a crucial role in providing structural integrity to cells and tissues. Nevertheless, in liver fibrosis, the normal regulation of the ECM is disrupted, resulting in the overproduction and deposition of ECM proteins due to persistent liver damage [4].

Heat shock proteins (HSPs) are essential for maintaining cellular homeostasis and serve as chaperones by aiding in the correct folding and assembly of newly synthesized proteins, as well as preventing protein aggregation. They play a crucial role in maintaining cellular equilibrium. Recent findings suggest that HSPs possess additional functions beyond their conventional role as molecular chaperones [5,6,7]. Specifically, they have been implicated in modulating cytokine induction and the inflammatory response, highlighting their significance in various biological processes. During fibrogenesis, several events take place, including the recruitment of inflammatory cells, the increased secretion of pro-fibrotic cytokines like transforming growth factor-β1 (TGF-β1), heightened apoptosis, oxidative stress, and the degradation of proteins through the proteasomal system. It is plausible that these events are associated with the involvement of HSPs [8].

HSP90 plays a vital role in the activation and stabilization of over 200 client proteins. It is indispensable for numerous cell signaling pathways. Recent research has proven that HSP90 also participates in the regulation of fibrotic disorders [9,10,11,12], including liver fibrosis [9,13]. Within this context, HSPs act as collagen-specific molecular chaperones, creating favorable conditions for collagen production [14]. The hallmark of fibrosis in various tissues and organs is the excessive production and deposition of collagen [15]. Hence, the expression and activity of HSP90 could be closely associated with the process of fibrogenesis and the progression of fibrosis. 

Inhibiting HSP90 has demonstrated effectiveness in mitigating fibroblast activation and disrupting the TGF-β signaling pathway, leading to a decrease in ECM production and collagen synthesis [16,17]. HSP90 inhibitors have pleiotropic effects, encompassing the degradation of specific proteins involved in various signaling pathways associated with inflammation, cell proliferation, and fibrogenesis [16,18,19]. Additionally, HSP90 inhibition has been shown to reduce oxidative stress and inflammation, thereby impacting the recruitment and activation of crucial immune cells like neutrophils and macrophages [20]. Notably, the inhibition of HSP90 has been observed to decrease the production of significant pro-inflammatory cytokines, including IL-1β, IL-6, CXCL10, and tumor necrosis factor (TNF), which are implicated in the progression of COVID-19. This reduction is likely due to the role of HSP90 in the activation pathway of IKK and nuclear transcription factor kappa B (NFκB) [21].

Furthermore, Studies have indicated that the administration of exogenous HSP90 can induce changes in the structure and composition of the ECM [22]. Furthermore, HSP90 plays a critical role in the activation and survival promotion of hepatic stellate cells (HSCs), the primary contributors to ECM production in liver fibrosis. Therefore, the survival of activated HSCs is heavily reliant on HSP90, highlighting a rationale for targeting HSP90 as a potential strategy to prevent liver fibrosis [8]. However, more investigations are needed to fully understand the mechanisms by which HSP90 regulates fibrogenic proteins and to develop effective therapies that target HSP90.

The 20S proteasome, previously known as the multicatalytic proteinase complex, consists of multiple peptidase activities that operate via a proteolytic mechanism involving a threonine active site. On the other hand, the 26S complex is an ATP-dependent protease responsible for the degradation of ubiquitinated proteins [23]. Moreover, it is a complex assembly that includes the 20S proteasome as one of its subunits [24]. Proteasomes and lysosomes are the two main cellular proteolytic systems. While lysosomes are crucial for breaking down endocytosed proteins, proteasomes, as non-lysosomal threonine proteases, serve as the primary protein degradation machinery in eukaryotes. It has been established that TGF-β receptor type-2 (TβRII), a protein of interest in the current research, can undergo degradation via the proteasome degradation system, either through ubiquitination-dependent or ubiquitination-independent pathways [25]. Hence, the 20S proteasome can degrade TβRII protein in an ATP-independent manner, without requiring ubiquitin. Therefore, targeting the proteasome could potentially be a therapeutic approach for fibrotic diseases, aiming to recognize and degrade fibrogenic proteins like TβRII. Yet, more studies are necessary to comprehensively unveil the mechanisms by which the proteasome regulates fibrogenic proteins and to develop effective therapeutic strategies targeting the proteasome.

HSP90 acts as a molecular chaperone that assists in the folding and stabilization of TβRII, thereby potentially regulating TGF-β1 signaling [26]. However, the specific effects of HSP90 inhibitors, such as STA9090, remain largely unknown. Unlike other HSP90 inhibitors, STA9090 is a synthetic small-molecule inhibitor that belongs to the resorcinol-based class that lacks the presence of the benzoquinone moiety, which is known to be associated with hepatotoxicity [27]. It is noteworthy for its exceptional effectiveness and safety characteristics, which include the absence of liver and eye toxicity [28,29]. Clinical studies conducted during phase I and II trials have demonstrated that STA9090, used as a single agent, is well tolerated with no consistent reports of hepatotoxicity. Additionally, the incidence of visual impairment, a common side effect of other HSP90 inhibitors, is notably low (<3%) with STA9090 [30,31]. The potential hepatotoxicity of STA9090 was investigated in male SD rats through a study involving the administration of repeated doses of the compound at 25, 50, and 75 mg/kg/day for 5 days. Notably, even at the highest dose of 75 mg/kg, surpassing the effective dosage range, no notable alterations in the levels of aspartate aminotransferase (AST) and alanine aminotransferase (ALT) enzymes were detected in the rats treated with STA9090. These findings align with the absence of enzymatic induction and revealed no discernible morphological alterations in the hepatocytes of animals treated with STA9090. Consequently, the unique pharmacokinetic characteristics and biological properties of STA9090 position it as a superior compound compared to earlier generations of HSP90 inhibitors [28,32].

In the current study, our primary objective was to enhance the destabilization of fibrogenic proteins that are clients of HSP90 through inhibition. Specifically, we were interested in investigating the effects of HSP90 inhibition on TβRII, a fibrogenic mediator that is known to interact with HSP90. When TβRII, an HSP90 client protein, is destabilized due to HSP90 inhibition, it can undergo degradation through the 20S proteasome pathway. By focusing on the HSP90-inhibition-induced destabilization of fibrogenic proteins, including TβRII, we aim to understand the mechanisms by which HSP90 inhibition can potentially disrupt fibrogenesis. This line of investigation has significant implications for developing therapeutic strategies targeting the development of fibrosis. Therefore, we seek to elucidate the impact of HSP90 inhibition using STA9090 on fibrogenic proteins, particularly TβRII, and its subsequent degradation via the 20S proteasome pathway when destabilized by HSP90 inhibition.

In this study, thioacetamide (TAA) was selected as the inducing agent for hepatocyte injury because it has the capability to mimic liver fibrosis and replicate the clinical characteristics of chronic liver disease [33]. The liver injury and damage caused by TAA in animal models resemble clinical liver disease, making it a valuable tool for studying liver fibrosis and assessing potential treatments [34]. TAA induces inflammation, oxidative stress, apoptosis, and extensive fibrosis, closely resembling the key features of liver fibrosis, which involve the accumulation of scar tissue and liver inflammation [35].

## 2. Results

### 2.1. Effect of STA9090 on Cell Viability

**Figure 1:**
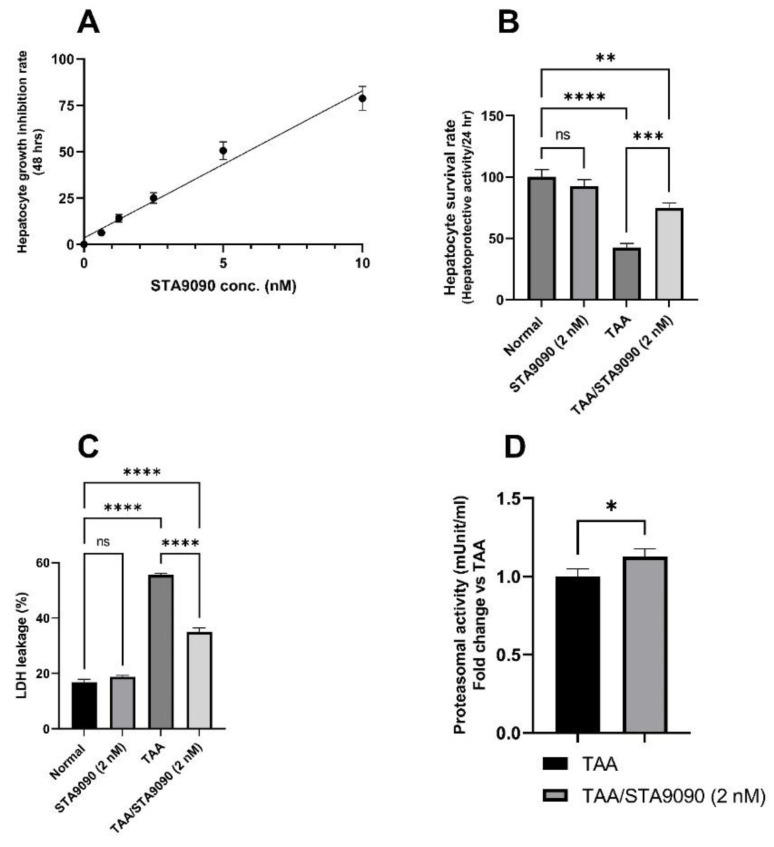
Effect of STA9090 on the hepatocyte growth inhibition rate (**A**), hepatocyte survival rate (**B**), LDH leakage (**C**), and proteasomal activity in the primary rat hepatocytes (**D**). (**A**) depicts the evaluation of the inhibitory effect of STA9090 on hepatocyte growth at various concentrations ranging from 0 to 10 nM. The CTC50 value was determined to be 5.85 nM and the concentration of 2 nM of STA9090 maintained over 80% viability of the primary rat hepatocytes. (**B**) demonstrates the hepatoprotective activity of STA9090 (2 nM) as measured by the survival rate of the hepatocytes. (**C**) presents the measurement of LDH leakage from hepatocytes into the culture media. (**D**) illustrates the effect of STA9090 treatment on 20S proteasomal activity in TAA-exposed hepatocytes. For group comparisons, a one-way ANOVA followed by Tukey’s post hoc test was conducted. An unpaired t-test was used to compare the means of two independent groups (proteasomal activity). The data are presented as the mean ± SD. Pairwise comparisons were used to determine the significance between the groups. *, *p* < 0.05; **, *p* < 0.01; ***, *p* < 0.001; ****, *p* < 0.0001. *n* = 6.

Based on the cytotoxicity testing, the inhibitory effect of STA9090 on hepatocyte growth was evaluated at various concentrations (0, 0.625, 1.25, 2.5, 5, and 10 nM), as shown in Figure 1A. The CTC50 value, which represents the concentration at which 50% of hepatocyte growth is inhibited, was determined to be 5.85 nM. Additionally, through linear regression analysis, it was observed that a concentration of 2 nM of STA9090 maintained over 80% viability of the primary rat hepatocytes. In reference to the study conducted by Zhou, et al. [36], which investigated the effects of STA9090 on SD rats, it was concluded that STA9090 administered at a dose of 20 mg/kg every other day is safe for hepatocytes. Therefore, based on the above-mentioned rationale of STA9090 dosing in the current study, this dosage was chosen for testing in vivo.

### 2.2. Effect of STA9090 on Hepatocyte Survival Rate

As shown in Figure 1B, the hepatoprotective activity of STA9090 (2 nM) was assessed by measuring the survival rate of the hepatocytes. It was observed that the hepatocyte survival rate was significantly increased in response to STA9090 (2 nM) treatment in the TAA-exposed hepatocytes compared to the untreated TAA-exposed hepatocytes (*p* < 0.001). This indicates that STA9090 treatment has a protective effect on hepatocytes, promoting their survival in the presence of TAA-induced toxicity.

### 2.3. Effect of STA9090 on LDH Release

As shown in Figure 1C, the hepatocytes treated with TAA displayed a significant increase in LDH leakage into the culture media compared to the normal hepatocytes. This indicates cellular damage and compromised membrane integrity. However, when the TAA-exposed hepatocytes were treated with STA9090, there was a significant decrease in LDH release compared to the untreated TAA-exposed hepatocytes. This suggests that STA9090 treatment can protect hepatocytes from TAA-induced damage and preserve their membrane integrity, leading to reduced LDH leakage.

### 2.4. Effect of STA9090 on 20S Proteasomal Activity (In Vitro)

As depicted in Figure 1D, the 20S proteasomal activity in the hepatocytes exposed to TAA was significantly increased in response to STA9090 treatment compared to the hepatocytes exposed to TAA alone.

### 2.5. Effect of STA9090 on Histological Features and the Necro-Inflammation Index

**Figure 2:**
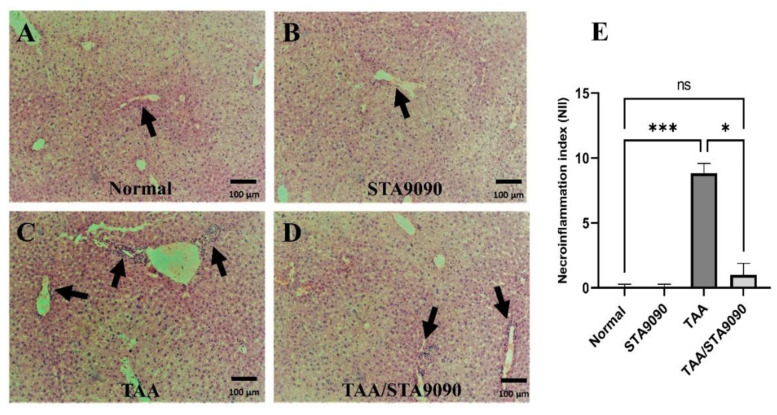
Effect of STA9090 on histological features and the necro-inflammation index: (**A**) shows liver sections from the Normal group, displaying normal architecture. The hepatocytes are arranged around the central veins, the portal tracts are intact (arrow), and the sinusoids are well preserved. In (**B**), the liver sections from the STA9090 group also exhibit a normal architecture, similar to the Normal group (arrow). In contrast, (**C**) demonstrates liver sections from the TAA-treated rats, which display distorted hepatic plates due to invading collagen, particularly around the portal tracts (arrows), resulting in portal–portal bridging fibrosis. Dilated sinusoids and infiltration of inflammatory cells are also observed. However, in the TAA/STA9090 group (**D**), the liver sections show reduced collagen deposition (arrow) compared to the TAA group. The necro-inflammation index is shown in (**E**). For group comparisons, the Kruskal–Wallis test was employed, followed by Dunn’s posthoc test. The data are presented as the median ± IQR. Pairwise comparisons were used to determine the significance between the groups. *, *p* < 0.05; ***, *p* < 0.001. *n* = 6.

The liver sections from the Normal group (Figure 2A) and the STA9090 group (Figure 2B) exhibited normal architecture with hepatocytes radiating out from central veins, intact portal tracts, and well-preserved sinusoids. In contrast, sections from the TAA-treated rats (Figure 2C) displayed distortion of hepatic plates caused by invading collagen, particularly around portal tracts, leading to the formation of portal–portal bridging fibrosis. Additionally, dilated sinusoids and infiltration of inflammatory cells were observed. However, in the TAA/STA9090 group, the liver sections showed attenuated collagen deposition (Figure 2D). Quantitative analysis revealed that the necro-inflammation index (Figure 2E) was significantly higher in the TAA group compared to the Normal group, indicating increased necrosis and inflammation. In contrast, the necro-inflammation index was significantly reduced in the TAA/STA9090 group compared to the TAA group. These findings suggest that STA9090 treatment attenuated collagen deposition and reduced necro-inflammation in the liver, indicating its potential antifibrotic and anti-inflammatory effects in this model.

### 2.6. Effect of STA9090 on the Area of Fibrosis

**Figure 3:**
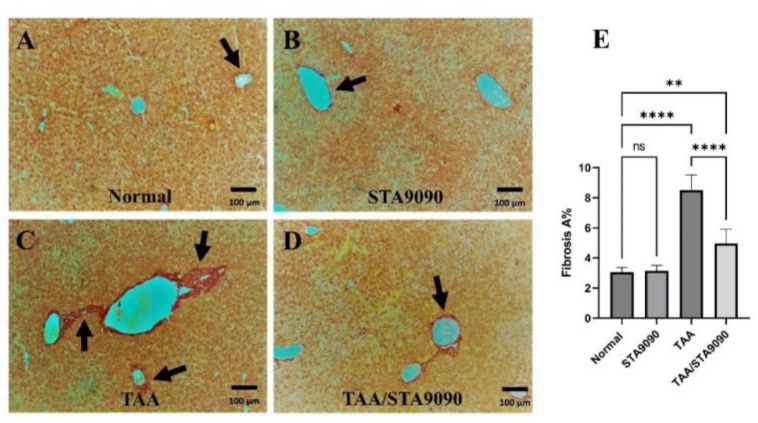
Effect of STA9090 on the area of fibrosis: (**A**) shows liver sections from the Normal group, displaying normal collagen deposition around the central veins and portal tracts (arrow). Similarly, (**B**) shows liver sections from the STA9090 group, exhibiting normal collagen deposition (arrow). In contrast, the liver sections from the TAA-treated rats (**C**) display excessive collagen invasion, particularly around the portal tracts, leading to the formation of portal–portal bridging fibrosis (arrows). However, in the TAA/STA9090 group (**D**), the liver sections show attenuated collagen deposition (arrow) compared to the TAA group. (**E**) depicts the quantitative analysis of the area % of fibrosis. For group comparisons, a one-way ANOVA followed by Tukey’s post hoc test was conducted. The data are presented as the mean ± SD. Pairwise comparisons were used to determine the significance between the groups. **, < 0.01; ****, *p* < 0.0001. *n* = 6.

The liver sections from the Normal group (Figure 3A) and STA9090 group (Figure 3B) exhibited normal collagen deposition around the central veins and portal tracts. In contrast, the liver sections from TAA-treated rats (Figure 3C) displayed excessive collagen invasion, particularly around the portal tracts, leading to the formation of portal–portal bridging fibrosis. However, in the TAA/STA9090 group, the liver sections showed attenuated collagen deposition (Figure 3D). As depicted in Figure 3E, quantitative analysis revealed that the area percentage (% area) of fibrosis was significantly higher in the liver sections from the TAA-treated rats compared to those from the normal rats. However, the % area of fibrosis was significantly reduced in the TAA/STA9090 group compared to the TAA group. These findings indicate that STA9090 treatment attenuated collagen deposition induced by TAA administration, suggesting its potential antifibrotic effects in this model.

### 2.7. Effect of STA9090 on ACTA2 Immunoexpression

**Figure 4:**
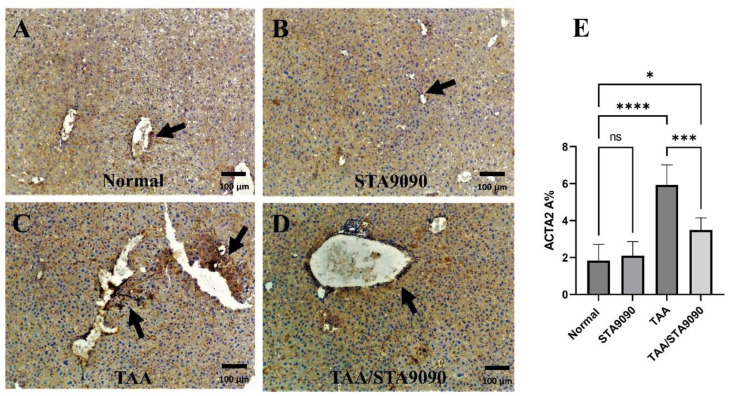
Effect of STA9090 on ACTA2 immunoexpression: (**A**) displays liver sections from the Normal group, showing normal ACTA2 immunoexpression surrounding the central veins and portal tracts (arrow). Similarly, (**B**) exhibits liver sections from the STA9090 group, displaying normal ACTA2 immunoexpression (arrow). In contrast, the liver sections from the TAA-treated rats (**C**) demonstrate excessive ACTA2 immunoexpression, particularly around the portal tracts (arrows). However, in the TAA/STA9090 group (**D**), the liver sections show attenuated ACTA2 immunoexpression (arrow) compared to the TAA group. (**E**) illustrates the quantitative analysis, revealing the area percentage (% area) of ACTA2 immunostaining. ACTA2 is the gene that encodes the alpha-smooth muscle actin protein. It is upregulated in fibrosis and helps drive the accumulation of myofibroblasts, which produce excess extracellular matrix. For group comparisons, a one-way ANOVA followed by Tukey’s post hoc test was conducted. The data are presented as the mean ± SD. Pairwise comparisons were used to determine the significance between the groups. * *p* < 0.05; *** *p* < 0.001; **** *p* < 0.0001. *n* = 6.

The liver sections from the Normal group (Figure 4A) and STA9090 group (Figure 4B) exhibited normal ACTA2 immunoexpression surrounding the central veins and portal tracts. In contrast, the liver sections from the TAA-treated rats (Figure 4C) displayed excessive ACTA2 immunoexpression, particularly around the portal tracts. However, in the TAA/STA9090 group, the liver sections showed attenuated ACTA2 immunoexpression (Figure 4D). As depicted in Figure 4E, the quantitative analysis revealed that the area percentage (% area) of ACTA2 immunostaining was significantly higher in the liver sections from the TAA-treated rats compared to those from the normal rats. However, the % area of ACTA2 immunostaining was significantly reduced in the TAA/STA9090 group compared to the TAA group. These findings indicate that STA9090 treatment mitigated the excessive ACTA2 expression induced by TAA administration, suggesting its potential antifibrotic effects in this model.

### 2.8. Effect of STA9090 on Survival Rate

**Figure 5:**
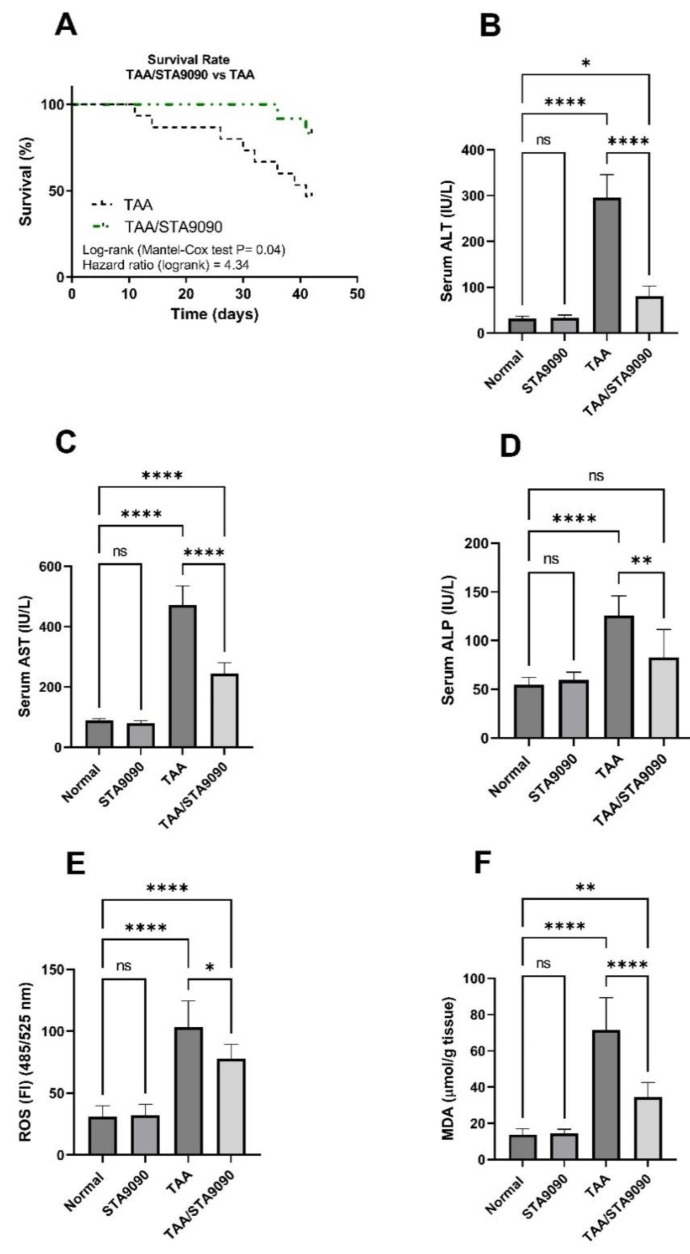
Effect of STA9090 on survival rate (**A**), ALT (**B**), AST (**C**), ALP (**D**), ROS (**E**), and MDA (**F**). A Kaplan–Meier survival plot was constructed and log-rank (Mantel–Cox test) was used for the determination of survival probability. For group comparisons, a one-way ANOVA followed by Tukey’s post hoc test was conducted. The data are presented as the mean ± SD. Pairwise comparisons were used to determine the significance between the groups. *, *p* < 0.05; **, < 0.01; ****, *p* < 0.0001.

The survival analysis was conducted by employing the log-rank (Mantel–Cox) test to compare the TAA-exposed group with the TAA/STA9090 group. The results of the analysis revealed a significant increase in the survival rate of TAA-exposed rats upon administration of STA9090 (Figure 5A) (*p* = 0.04). The hazard ratio (log-rank) was calculated as 4.34, indicating a 4.34-fold higher likelihood of survival in the TAA/STA9090 group compared to the TAA-exposed group. The 95% confidence interval (CI) for the hazard ratio ranged from 1.256 to 15.02, suggesting a significant improvement in survival outcomes with the STA9090 treatment. It is important to note that in our study, a necropsy was not conducted to determine the precise cause of death. However, a lack of liver function, coagulopathy, and the accumulation of toxins induced by TAA can contribute to the eventual death of experimental animals.

### 2.9. Effect of STA9090 on Liver Function Enzymes

The levels of the liver function enzymes ALT (Figure 5B), AST (Figure 5C), and ALP (Figure 5D) exhibited a significant increase in response to TAA administration compared to their respective normal levels. However, treatment with STA9090 at the selected dosage significantly decreased these enzyme levels in the TAA/STA9090 rats compared to the rats administered with TAA alone. The elevation of ALT, AST, and ALP indicates liver damage or dysfunction. The observed reduction in these enzyme levels following STA9090 administration at 20 mg/kg suggests the potential of STA9090 to mitigate liver injury and enhance liver function in the TAA-induced model.

### 2.10. Effect of STA9090 on ROS and MDA levels

In comparison with the normal rats, the TAA group demonstrated a significant elevation in ROS production (Figure 5E) and MDA levels (Figure 5F). Conversely, treatment with STA9090 resulted in a significant reduction in ROS and MDA levels compared to the TAA group. ROS are highly reactive molecules that can cause cellular damage and induce oxidative stress. MDA is a by-product of lipid peroxidation and serves as a marker of oxidative stress and lipid damage. The increased levels of ROS and MDA observed in the TAA group indicate heightened oxidative stress and lipid peroxidation. However, STA9090 treatment, at the selected dosage, effectively mitigated these levels, suggesting its ability to alleviate oxidative stress and lipid damage in the liver.

### 2.11. Effect of STA9090 on GSH, SOD, and CAT

**Figure 6:**
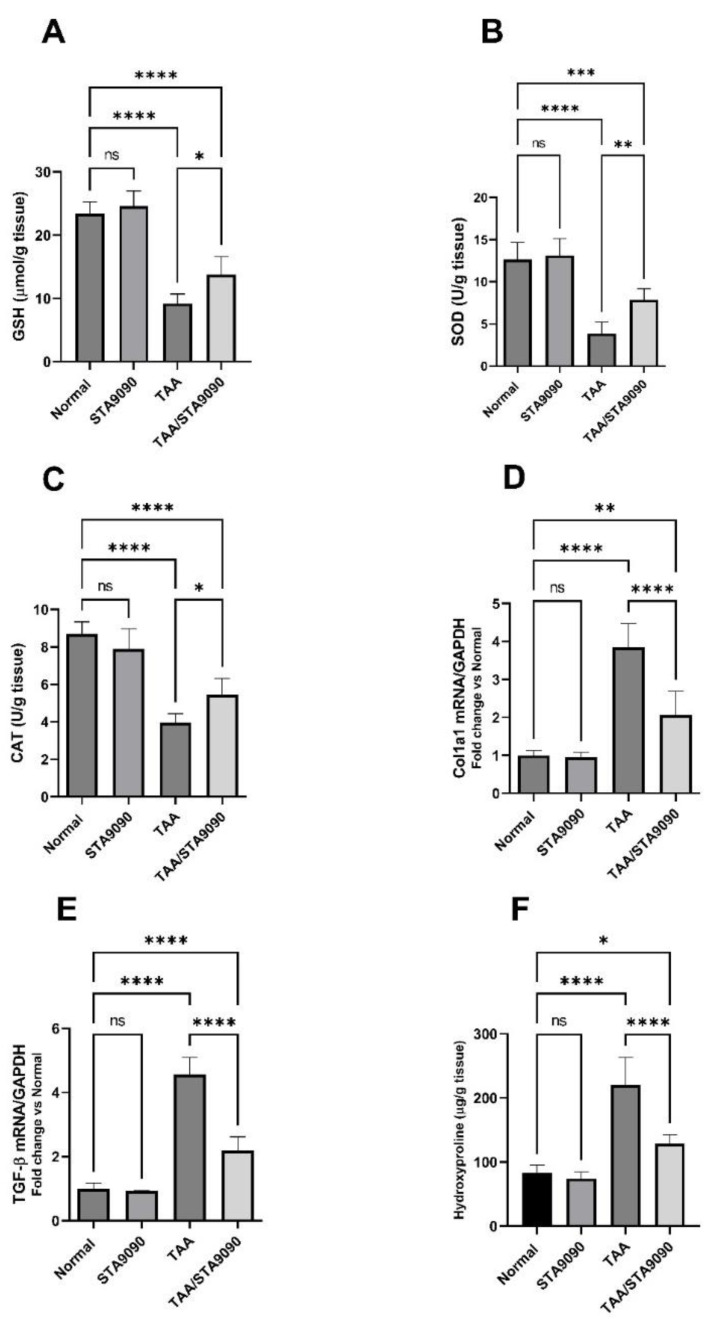
Effect of STA9090 on GSH (**A**), SOD (**B**), CAT (**C**), Col1a1 mRNA (**D**), TGF-β mRNA (**E**), and hydroxyproline (**F**). For group comparisons, a one-way ANOVA followed by Tukey’s post hoc test was conducted. The data are presented as the mean ± SD. Pairwise comparisons were used to determine the significance between the groups. *, *p* < 0.05; **, < 0.01; ***, *p* < 0.001; ****, *p* < 0.0001. *n* = 6.

In comparison with the normal rats, the TAA group demonstrated a significant reduction in the levels of GSH (Figure 6A), SOD (Figure 6B), and CAT (Figure 6C). In contrast, treatment with STA9090 resulted in a noteworthy elevation in their levels compared to the TAA group. GSH is a vital antioxidant molecule, while SOD and CAT are enzymes involved in cellular antioxidant defense mechanisms. The decrease in GSH, SOD, and CAT levels observed in the TAA group indicates the presence of oxidative stress and impaired antioxidant capacity. However, treatment with STA9090 effectively reversed these effects, leading to an increase in GSH, SOD, and CAT levels. This increase suggests the restoration of antioxidant defense mechanisms in the liver [37] due to STA9090 treatment at the selected dosage.

### 2.12. Effect of STA9090 on Col1a1 and TGF-β mRNA Expression

In comparison with the normal rats, the TAA group displayed a significant increase in the mRNA levels of Col1a1 (Figure 6D) and TGF-β (Figure 6E). However, administration of STA9090 resulted in a significant reduction in their expression levels when compared to the TAA group. The upregulation of Col1a1 mRNA indicates enhanced collagen production, which is associated with fibrosis. Similarly, elevated TGF-β mRNA expression suggests activation of pro-fibrotic signaling pathways. Nevertheless, treatment with STA9090 effectively reversed these effects, leading to a decrease in Col1a1 and TGF-β mRNA expression. This reversal may contribute to the antifibrotic effects of STA9090 in this model.

### 2.13. Effect of STA9090 on Hydroxyproline Liver Content

In comparison with the normal rats, the TAA group demonstrated a significant rise in hydroxyproline content (Figure 6F), while treatment with STA9090 led to a significant decline in its levels compared to the TAA group. Hydroxyproline is an amino acid primarily present in collagen, and its content is commonly utilized as an indicator of total collagen. Higher levels of hydroxyproline indicate increased collagen deposition and greater fibrosis within the tissue.

### 2.14. Effect of STA9090 on HSP90 and HSP70 

**Figure 7:**
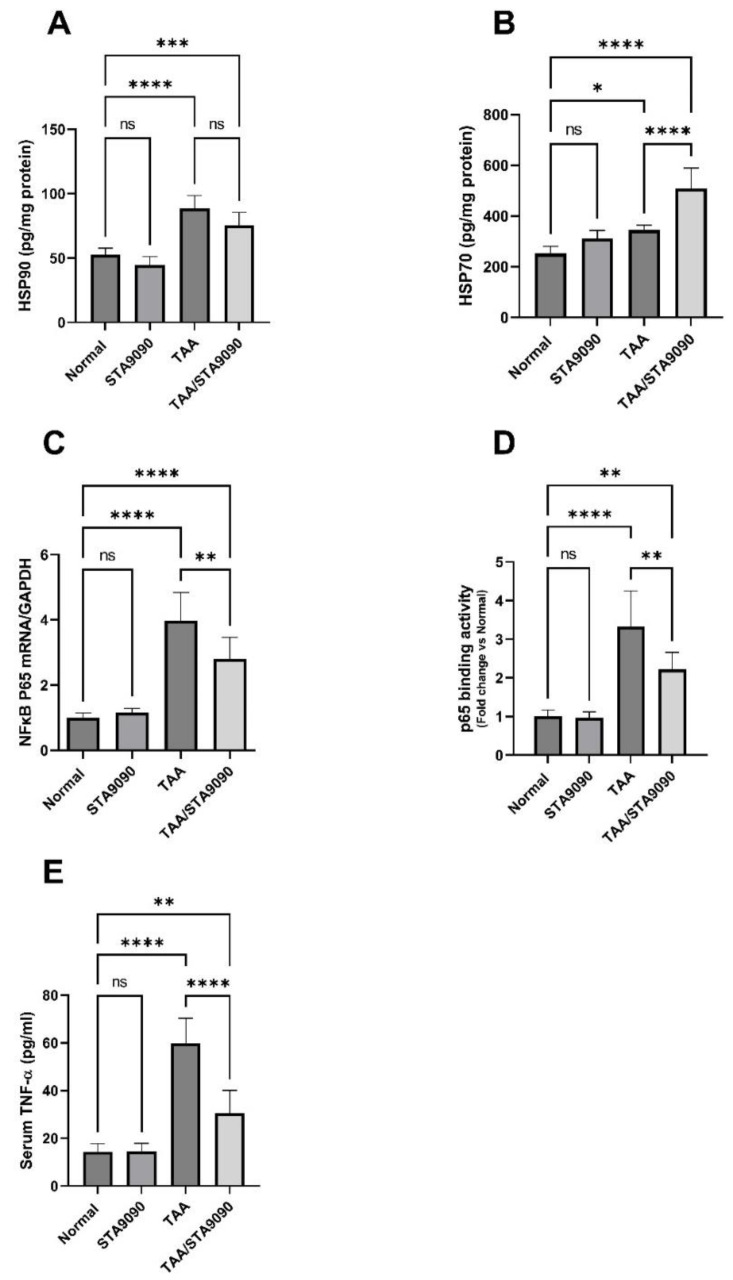
Effect of STA9090 on HSP90 (**A**), HSP70 (**B**), NFκB p65 mRNA (**C**), P65 binding activity (**D**), and TNF-α (**E**). For group comparisons, a one-way ANOVA followed by Tukey’s post hoc test was conducted. The data are presented as the mean ± SD. Pairwise comparisons were used to determine the significance between the groups. *, *p* < 0.05; **, < 0.01; ***, *p* < 0.001; ****, *p* < 0.0001. *n* = 6.

The administration of TAA led to a significant increase in the levels of HSP90 and HSP70, as depicted in Figure 7A,B, respectively, when compared to the normal control group. Notably, the administration of STA9090 exhibited a significant elevation in HSP70 levels when compared to the TAA group. However, there was no significant impact on HSP90 levels observed with STA9090 treatment. Notably, HSP70 is commonly acknowledged as a reliable marker of HSP90 inhibition in biological systems, and its increase is likely attributable to the impact of STA9090 treatment.

### 2.15. Effect of STA9090 on NFκB p65 mRNA Expression, p65 DNA Binding Activity, and the Levels of TNF-α

In comparison with the normal rats, the TAA group demonstrated a significant elevation in the mRNA expression of NFκB p65, the DNA binding activity of p65, and the levels of TNF-α (Figure 7C–E, respectively). Conversely, treatment with STA9090 resulted in a significant decrease in these levels compared to the TAA group. These findings indicate that TAA administration led to upregulation of NFκB expression and activity, as well as an increase in TNF-α levels. However, STA9090 treatment effectively reversed these effects, suggesting its potential anti-inflammatory effects through the inhibition of NFκB. It is worth noting that TNF-α is one of the key target genes regulated by NFκB [38,39].

### 2.16. Effect of STA9090 on TGF-β and the Ratio of MMP-9/TIMP-1

**Figure 8:**
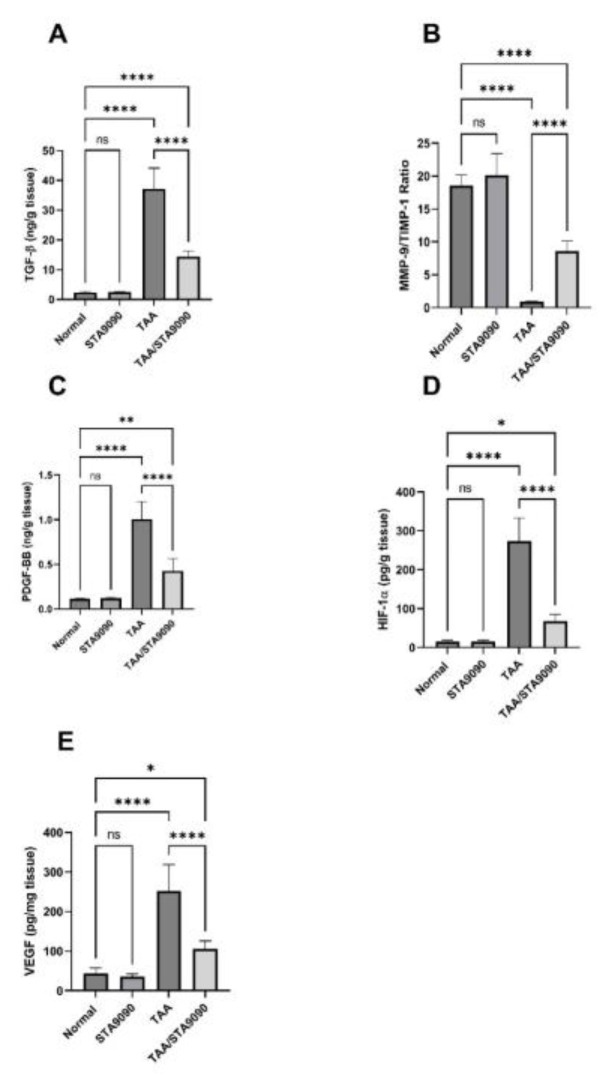
Effect of STA9090 on TGF-β (**A**), MMP-9/TIMP-1 ratio (**B**), PDGF-BB (**C**), HIF-1α (**D**), and VEGF (**E**). For group comparisons, a one-way ANOVA followed by Tukey’s post hoc test was conducted. The data are presented as the mean ± SD. Pairwise comparisons were used to determine the significance between the groups. *, *p* < 0.05; **, *p* < 0.001; ****, *p* < 0.0001. *n* = 6.

In comparison with the normal rats, the TAA group presented a significant rise in the levels of TGF-β and the ratio of MMP-9/TIMP-1 (Figure 8A,B, respectively). However, treatment with STA9090 led to a significant decline in the levels of TGF-β and the ratio of MMP-9/TIMP-1 compared to the TAA group. These findings suggest that TAA administration activated profibrogenic TGF-β signaling and disrupted the homeostasis of the ECM, as evidenced by elevated TGF-β levels and an imbalanced MMP-9/TIMP-1 ratio. Conversely, STA9090 treatment significantly reversed these effects, which may contribute to its antifibrotic mechanisms. TGF-β is a cytokine that plays a pivotal role in activating hepatic stellate cells and promoting the production of collagen, thus driving the progression of liver fibrosis. MMP-9 is an enzyme responsible for the degradation of ECM proteins, while TIMP-1 serves as an inhibitor of MMP-9. An elevated MMP-9/TIMP-1 ratio indicates a shift towards increased ECM degradation.

### 2.17. Effect of STA9090 on PDGF-BB, HIF-1α, and VEGF

In comparison with the normal rats, the TAA group demonstrated a significant rise in the levels of PDGF-BB, HIF-1α, and VEGF (Figure 8C–E, respectively). However, treatment with STA9090 led to a significant decline in these levels compared to the TAA group. The elevated levels of these profibrotic and pro-angiogenic mediators in the TAA group suggest the promotion of fibrogenesis and angiogenesis. Conversely, STA9090 treatment effectively counteracted these effects by reducing the levels of PDGF-BB, HIF-1α, and VEGF, which may contribute to its antifibrotic effects in this model. PDGF-BB plays a role in fibrosis progression by activating hepatic stellate cells [40]. VEGF, on the other hand, stimulates angiogenesis, which can contribute to fibrosis [41]. HIF-1α, a transcription factor, binds to and activates the VEGF gene promoter, thereby stimulating VEGF transcription. Additionally, HIF-1α stabilizes VEGF mRNA, leading to increased translation of the VEGF protein [42]. It is important to mention that HIF-1α is an HSP90 client protein. HSP90 binding maintains HIF-1α in its active conformation and prevents its degradation. Inhibition of HSP90 by STA9090 disrupts this interaction, resulting in the instability and rapid degradation of HIF-1α [43].

### 2.18. Effect of STA9090 on 20S Proteasomal Activity in the Liver Tissue

**Figure 9:**
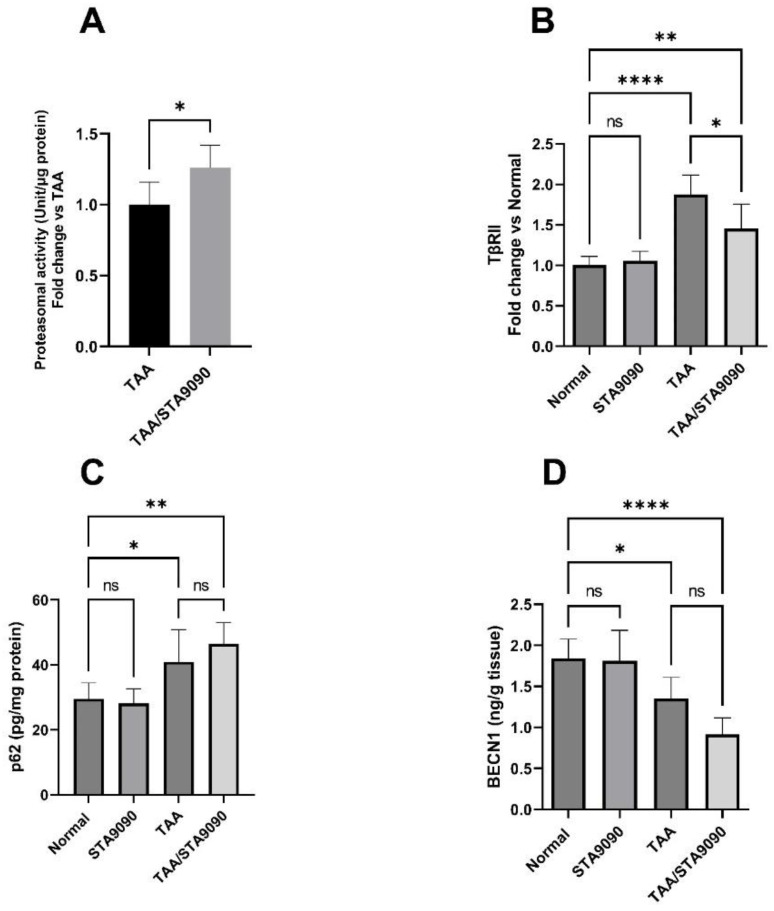
Effect of STA9090 on proteasomal activity in liver tissue (**A**), TβRII (**B**), VEGFR2 (**C**), p62 (**D**). For group comparisons, a one-way ANOVA followed by Tukey’s post hoc test was conducted. The data are presented as the mean ± SD. Pairwise comparisons were used to determine the significance between the groups. * *p* < 0.05; ** < 0.01; **** *p* < 0.0001. *n* = 6.

As illustrated in Figure 9A, treatment with STA9090 led to a significant increase in 20S proteasomal activity in the livers of rats exposed to TAA when compared to the group not treated with STA9090. This finding indicates that STA9090 treatment significantly enhanced 20S proteasomal activity in the fibrotic livers of the TAA-exposed rats. The upregulation of this protein degradation pathway likely serves as one mechanism through which STA9090 exerts its antifibrotic effects by facilitating the breakdown of profibrotic proteins.

### 2.19. Effect of STA9090 on TβRII 

In comparison with the normal rats, the TAA group showed a significant rise in the levels of TβRII (Figure 9B). However, treatment with STA9090 led to a significant decline in these levels in comparison with the TAA group. These findings suggest that TAA administration activated TGF-β to contribute to fibrosis. Conversely, STA9090 treatment effectively reversed these effects by reducing the levels of TβRII, which may explain its antifibrotic effects in this model. It is important to highlight that TβRII expression levels serve as an indicator of the activity of the pro-fibrotic TGF-β pathway. TGF-β signaling, mediated by TβRII, plays a crucial role in promoting the activation of hepatic stellate cells and collagen production, thereby driving liver fibrosis. 

The observed decline in TβRII levels can be attributed to the increased 20S proteasomal activity in the context of HSP90 inhibition caused by STA9090. HSP90 inhibition destabilizes its client proteins, including the membrane receptors TβRII. As a result, the 20S proteasome degrades these proteins at a higher rate. This proteasome-mediated degradation of profibrotic proteins represents one potential mechanism through which STA9090 exerts its anti-fibrotic effects. It is worth noting that TβRII can be degraded via the ubiquitin-dependent and -independent proteasome system, while lysosomal degradation also contributes to its turnover, albeit to a lesser extent.

### 2.20. Effect of STA9090 on p62 and BECN1

The administration of TAA led to a notable rise in the levels of p62 (Figure 9C) and a significant decrease in the level of BECN1 (Figure 9D) compared to the corresponding normal values. These findings indicate the suppression of autophagy. Interestingly, in the TAA/STA9090 group, STA9090 treatment had no significant impact on these levels in comparison with the TAA group. This suggests that STA9090 treatment had a limited impact on the autophagy process in the TAA-induced fibrotic model. Importantly, elevated p62 levels and reduced BECN1 levels, which serve as markers of autophagy, indicate a hindrance in the flow of autophagic activity. This finding potentially rules out the involvement of the autophagy–lysosomal pathway in the degradation of TβRII.

## 3. Discussion

Liver fibrosis is a progressive condition characterized by inflammation and the accumulation of fibrous tissue resulting from chronic liver injury. If left untreated, it can lead to liver cirrhosis, liver failure, or even liver cancer. Despite advancements in research and treatment, there is still a significant knowledge gap regarding the underlying mechanisms of liver fibrosis. Consequently, there is a critical need to develop a deeper understanding of this complex process in order to identify more effective strategies for managing and treating liver fibrosis. By creating new opportunities for research and innovation, we can pave the way for the improved management of liver fibrosis [44].

In the present study, our in vitro testing revealed that STA9090 increased the hepatocyte survival rate and decreased LDH leakage. Remarkably, STA9090 increased 20S proteasomal activity in TAA-exposed hepatocytes. Additionally, we determined that STA9090 at a dose of 20 mg/kg is safe for hepatocytes to be tested in vivo. In addition, our in vivo testing revealed that inhibiting HSP90 with STA9090 has shown promise in reducing oxidative stress, improving liver function, and increasing the survival of TAA-exposed rats. STA9090 exerted pleotropic effects by targeting proteins involved in inflammation and constrained the production of collagen leading to improved microscopic features of the rat livers.

The administration of STA9090, an HSP90 inhibitor, has been found to enhance the activity of the 20S proteasome, leading to the degradation of TβRII, a fibrogenic client protein of HSP90. The process involves two steps. Firstly, STA9090-induced HSP90 inhibition destabilizes TβRII. Secondly, the destabilized TβRII is then degraded through the activated 20S proteasome facilitated by STA9090. Therefore, HSP90 inhibition with STA9090 not only destabilizes the fibrogenic HSP90 client protein TβRII but also promotes its degradation through the activation of the 20S proteasomal degradation system. Specifically, the proteasome plays a crucial role in inhibiting TGF-β signaling by interacting with TβRII and facilitating its degradation. This mechanism is significant in controlling and attenuating the effects of the TGF-β signaling pathway. This novel finding suggests that targeting HSP90 with STA9090 could be a potential approach to prevent the development of liver fibrosis.

Autophagy is a tightly controlled cellular mechanism that plays a crucial role in the breakdown and recycling of cellular components, such as organelles and proteins. It involves the formation of a double-membrane vesicle called an autophagosome, which engulfs the targeted material and fuses with a lysosome to form an autolysosome. Within the autolysosome, lysosomal enzymes degrade the enclosed material. Lysosomes play a crucial role in the degradation of cellular components that are no longer needed or have become damaged. However, in the present study, it was observed that STA9090 does not affect the autophagy degradation system. This finding suggests that the degradation of the fibrogenic protein TβRII induced by STA9090 is independent of the autophagy pathway. It is noteworthy that the turnover of TβRII involves not only the proteasomal degradation pathway but also the lysosomal degradation pathway, albeit to a lesser extent. However, our findings specifically exclude the involvement of the autophagy–lysosomal degradation pathway in the degradation of TβRII. Instead, our results emphasize the significant role of the proteasomal degradation pathway in regulating the turnover of TβRII.

Another notable HSP90 client protein is HIF-α, which was found to be inhibited by STA9090 in our study. Under normoxic conditions, HIF-α is rapidly degraded by the ubiquitin–proteasome system. The mechanisms underlying the degradation of HIF-1α have been elucidated, involving the hydroxylation of specific amino acid residues by prolyl-hydroxylase domain (PHD)-containing proteins. This hydroxylation leads to the binding of HIF-α to the tumor suppressor von-Hippel–Lindau protein (pVHL), ultimately resulting in proteasomal degradation [45]. Further investigations are required to confirm whether STA9090 can regulate the ubiquitin–proteasome system responsible for HIF-1α degradation. However, based on the available data, our findings suggest that HSP90 inhibition by STA9090 destabilizes HIF-1α, making it susceptible to degradation through the ubiquitin–proteasome system, ultimately leading to reduced transcription of VEGF as one of its target genes. VEGF is an angiogenic and fibrogenic factor whose expression is regulated by HIF-1α [46].

The present study highlights the potential of STA9090 to exhibit anti-inflammatory and antifibrotic effects, which are believed to be connected to its ability to deactivate the NFκB signaling pathway. NFκB, a transcription factor, is involved in the production of pro-inflammatory cytokines and other molecules that contribute to tissue damage and fibrotic processes. Consequently, hindering NFκB signaling has the potential to mitigate the inflammatory response and prevent the development of fibrosis. The NFκB signaling pathway is involved in regulating specific fibrogenic markers, including TGF-β, TIMP-1, MMP-9, and PDGF-BB [47,48].

Upon exposure to various stimuli such as cellular stress and chemical damage, cells upregulate the expression of HSP70 to prevent protein aggregation [49]. Notably, research has revealed that HSP70 has a negative impact on epithelial–mesenchymal transition (EMT), a key process involved in fibrosis [50]. The deletion of HSP70 stimulates the signaling cascade of TGF-β activation, consequently increasing the production of ECM [7]. Additionally, the deficiency of HSP70 has been linked to the development of fibrosis, suggesting that interventions targeting the restoration of normal HSP70 expression could serve as a promising therapeutic approach [10]. In our study, STA9090 treatment led to the induction of HSP70 expression, which serves as a biological marker of HSP90 inhibition. Inhibition of HSP90 triggers the upregulation of HSP70, which plays a crucial role in protecting cells from stress-induced damage by facilitating protein refolding or promoting their degradation. Therefore, the elevation in HSP70 expression levels following HSP90 inhibition can be utilized as a reliable marker of HSP90 inhibition, as it represents a downstream effect of this inhibition and indicates the cellular stress response triggered by HSP90 inhibition [51,52]. Although initial findings indicate that HSP70 could have a protective effect against liver fibrosis, our comprehension of the underlying molecular mechanisms remains incomplete. To definitively determine the role of HSP70 and its potential as a therapeutic target, further well designed studies are necessary to fill the significant gaps in our knowledge.

The severity of liver fibrosis can depend on various factors, including the amount of insult and the duration of exposure which can contribute to different levels of fibrosis severity. In this context, we highlight the importance of considering the dose and duration of exposure to fully comprehend the relationship between the intervention and the severity of fibrosis in future work in order to provide a more comprehensive evaluation of the effect of STA9090 on the development of liver fibrosis.

## 4. Methods

### 4.1. Isolation of Primary Rat Hepatocytes

The process of isolating rat hepatocytes from a 220 g male SD rat (TBRI, Egypt), was performed according to a two-step collagenase perfusion method. First, the rats were anesthetized as described [53]. Following a midline incision to expose the portal vein, a cannula (18-gauge) was inserted into the vein and connected to a tube carrying a perfusate. Second, the perfusion process was initiated by introducing a warm (37 °C) HBSS without calcium, which included MgCl2 (0.9 mmol/L), EDTA (0.5 mmol/L), and HEPES (25 mmol/L), at a flow rate of 10 mL/min. After confirming the successful insertion of the cannula, an incision was made in the inferior vena cava to enable fluid outflow, and the flow rate was raised to 25 mL/min. Subsequently, a second warm (37 °C) perfusion buffer, which was HBSS containing calcium and magnesium, along with HEPES (25 mmol/L) and collagenase type IV (10 g/L), was introduced while maintaining the flow. To promote liver swelling and facilitate the dissociation of hepatocytes, pressure-release cycles were applied to the inferior vena cava using cotton swabs. Once the collagenase perfusion was finished, the liver was carefully dissected and moved to a sterile setting within a tissue cell culture hood. It was then gently spread out in a sterile petri dish that contained warm (37 °C) DMEM and passed through a Nylon filter to eliminate any undigested tissues and debris. The obtained cells were placed in suspension in DMEM and then subjected to centrifugation at 50× *g* for 3 min at 4 °C. The supernatant was carefully removed, and this process was repeated. Following the final wash, the supernatant was discarded, and the cells were suspended in a combination of 25 mL DMEM and 25 mL Percoll solution in PBS. Following centrifugation at 200× *g* for 10 min at 4 °C, the non-viable cells were removed from the top, while the viable cells settled at the bottom and were subsequently resuspended in 20 mL of warm (37 °C) DMEM. This procedure was repeated twice. The cells were then counted using a hemocytometer, and their viability was assessed using the Trypan blue dye exclusion method.

#### Assessment of Cytotoxicity and Hepatocyte Viability

After the isolation process, the hepatocytes were evenly distributed and placed in a 6-well plate at a density of 4 × 10^5^ cells per well. They were then kept in a humidified CO_2_ incubator for 20 h, with DMEM being used as the culture medium, which was replaced after 3 h. Once the initial culture period was completed, the hepatocytes were sub-cultured and seeded in a 96-well culture plate at a density of 2 × 10^4^ cells per well. The culture medium used for this step contained DMEM supplemented with 10% fetal bovine serum (FBS), 100 IU/mL of penicillin, 100 µg/mL of streptomycin, and 2 mM glutamine. Subsequently, the cells were placed in an incubator at a temperature of 37 °C and a humidified atmosphere composed of 5% CO_2_, 74% N_2_, and 21% O_2_ for one day. Following this 24-h culture period, the supernatants were removed, and the culture media were replaced with a fresh medium containing 0.5% dimethyl sulfoxide (DMSO) along with various concentrations of STA9090 (0, 1.25, 2.5, 5, or 10 nM). The cells were then incubated with this new medium for a period of 2 days. To evaluate the cytotoxicity, a colorimetric assay utilizing 3-(4,5-dimethyl-2-thiazolyl)-2,5-diphenyl-2-H-tetrazolium bromide (MTT) was conducted. In this assay, 20 µL of MTT solution (0.5 mg/mL in DMEM) was introduced to each well and incubated for a duration of 4 h. The resulting formazan crystals were dissolved by adding 50 µL of DMSO, and the plates were further incubated at 37 °C for 30 min while being agitated. The optical density of each well was assessed at a wavelength of 595 nm using a microplate spectrophotometer reader. To evaluate the cytotoxicity, the percentage growth inhibition was calculated by normalizing the percentage of viable cells to the untreated group (zero concentration). Each concentration of STA9090 was tested in triplicate. The concentration that maintained hepatocyte viability above 80% after 48 h was considered safe for further investigation into its hepatoprotective activity.

### 4.2. Determination of the In Vitro Hepatoprotective Activity of STA9090 

The performed MTT assay was used to determine the appropriate dose of STA9090 for the investigation of its hepatoprotective activity in primary rat hepatocytes insulted with TAA (100 mM). The hepatocytes were sub-cultured and seeded in a 96-well plate at a density of 2 × 10^4^ cells per well. In the Normal group, the hepatocytes were exposed to DMEM (100 µL) supplemented with 0.5% DMSO as the culture medium for a period of 24 h. On the other hand, the STA9090 group was treated with the culture medium containing 0.5% DMSO along with STA9090 (2 nM) for the same duration. The TAA group was treated with culture media containing TAA (100 mM) (Sigma-Aldrich, Burlington, MA, USA) and 0.5% DMSO for the same time course. The STA9090/TAA group received the culture media containing TAA (100 mM), DMSO (0.5%), and STA9090 (2 nM) for the same time course. Following each time course which was performed in triplicate, the media were removed, and the MTT assay was conducted to estimate the percentage of cell viability. Additionally, another set of experimental groups was established, where hepatocytes were cultured for 24 h in 6-well culture plates at a density of 4 × 10^5^ cells per well. In this set, all cells were harvested for LDH leakage assay. The groups under investigation included the Normal (control group), STA9090, TAA, and STA9090/TAA groups. 

### 4.3. Determination of the Lactate Dehydrogenase (LDH) Leakage in Culture Media

The evaluation of TAA-induced hepatotoxicity involved assessing the activity of lactate dehydrogenase (LDH) using a commercially available kit from Sigma-Aldrich. Following incubation, the culture medium was collected and centrifuged at 300× *g* for 5 min. The resulting supernatant was then utilized to measure the LDH levels present in the culture medium. To measure the levels of LDH in the whole-cell lysate, the remaining cells were treated with a 10 mM phosphate buffer (pH 7.4) containing 1% (*w*/*v*) Triton X-100. The cell suspension was homogenized by passing it through a 27-gauge needle and subsequently centrifuged for 10 min at 800× *g* and 4 °C. The obtained supernatant fraction, which represented the whole-cell lysate, was carefully transferred to a new tube. The levels of LDH in both the culture medium and the whole-cell lysate were measured at a wavelength of 450 nm. To determine the percentage of LDH released into the media, the following formula was used: Media released LDH % = LDH_media_/(LDH_media_ + LDH_whole-cell lysate_) × 100.

### 4.4. Measurement of the Proteasomal Activity in Hepatocytes In Vitro

Isolated hepatocytes were cultured in 6-well culture plates for 48 h at a density of 4 × 10^5^ cells per well. In this experiment, cells (2 × 10^6^) were collected for the measurement of proteasomal activity. The experimental groups consisted of the TAA group (treated with 100 mM TAA + 0.5% DMSO) and the TAA/STA9090 group (treated with 100 mM TAA + 0.5% DMSO + 2 nM STA9090). The measurement of proteasomal chymotrypsin-like activity was conducted using an Abcam proteasomal activity kit, following the instructions provided by the manufacturer. To begin, proteasomes were extracted from the hepatocytes using a lysis buffer containing 0.5% NP-40 (a non-ionic detergent from Sigma) in PBS after hepatocytes were homogenized by pipetting up and down a few times. The samples were then centrifuged at 16,000× *g* for 15 min at 4 °C, and the protein concentration of each sample was determined using the BCA protein assay kit. Equal protein concentrations of cell lysates were incubated at 37 °C in two separate wells, one with the proteasomal substrate Succ-LLVY-AMC (which produces highly fluorescent AMC when proteolytic activity is present) and the other without the proteasome inhibitor MG-132 (serving as a negative control). After incubating the plate for 25 min, the resulting fluorescence, based on the cleaved AMC, was measured using a fluorometric plate reader at excitation/emission wavelengths of 350 nm/440 nm, respectively. Following an additional 30-min incubation at 37 °C, the plates were analyzed again to determine the change in relative fluorescence units for each sample. To isolate the proteasome activity from other protease activities in the samples, the fluorescence value of the sample treated with MG-132 was subtracted from the sample without MG-132 treatment. Proteasome activity was calculated based on the amount of proteasome activity required to generate 1.0 pmol of AMC per minute at 37 °C, with 1 mUnit/mL of proteasome activity defined as such. Finally, the obtained values were normalized to the proteasome activity of TAA-treated hepatocytes, which served as the control. The data are presented as a fold change of the control samples, with the control samples set to 1. Three independent experiments were conducted, with each sample assayed in duplicate.

### 4.5. Animals

Male adult SD rats weighing between 210–220 g were procured from TBRI for the research study. The rats were kept in standard housing conditions. A period of 2 weeks was allotted for the rats before starting the procedures. The treatment and handling of the animals adhered to the guidelines established by the FPDUREC and were granted approval under the identification number 16/2022,3.

### 4.6. Study Design

SelleckChem STA9090 (Houston, TX, USA) was acquired and dissolved in a DRD solution. This solution consisted of 10% DMSO, 18% Cremophor RH 40, 3.6% dextrose, and 68.4% water [54]. It is noteworthy that all animal groups received the same dosing schedule and were administered the vehicle, which was prepared using the aforementioned ingredients. In this study, the rats were randomly assigned to four groups (Table 1): the Normal group (*n* = 8) served as the control, the STA9090 group (*n* = 8) received STA9090 (20 mg/kg, every other day, intraperitoneal) as the drug control, the TAA group (*n* = 15) received TAA (Sigma) at a dose of 150 mg/kg, twice a week, intraperitoneal for 6 weeks, and the TAA/STA9090 group (*n* = 12) received both TAA and STA9090 (20 mg/kg, every other day, intraperitoneal) for 6 weeks. Before injection, TAA was dissolved in 0.9% normal saline, resulting in a total volume of 1 mL per rat, approximately one hour prior to administration. At the end of the sixth week, blood samples were collected from the retro-orbital plexus, and the rats were decapitated following anesthesia with a combination of 87.5 mg/kg ketamine and 12.5 mg/kg xylazine.

### 4.7. Rational of STA9090 Dosing

Zhou, et al. [36] conducted a study to investigate the effects of STA9090 on the SD rats. They administered STA9090 intravenously at a dose of 20 mg/kg and measured a plasma concentration of 0.08 µM after 6 h. The authors also determined that the mean unbound plasma fraction, representing the portion of STA9090 available for the on-target effects, was 2.5%. Additionally, the half-life (t_1/2_) of STA9090 was estimated to be around 6 h. Based on these findings, we extrapolated that after one half-life, the unbound plasma concentration of STA9090 would be approximately 2 nM. Referring to our conducted in vitro experiment we observed that a concentration of 2 nM of STA9090 maintained over 80% viability of primary rat hepatocytes. Considering these results, we concluded that STA9090 at a dose of 20 mg/kg every other day is safe for hepatocytes. Therefore, this dosage was chosen for testing in vivo in the current study.

### 4.8. H&E Staining and the Determination of the Necro-Inflammation Index (NII) 

The tissue samples underwent fixation in formalin, followed by embedding in paraffin blocks and sectioning into 4 μm-thick slices. These sections were then subjected to standard procedures for H&E staining [44,55]. Following staining, the liver sections were examined using a microscope. To quantify necro-inflammation, the modified Ishak necro-inflammation index [56] was employed. The severity of inflammation was assessed by summing up the scores from four categories of microscopic features, with higher scores indicating a more pronounced deviation from the normal control.

### 4.9. Sirius Red Staining and Determination of the Fibrosis Area %

The formalin-fixed tissue specimens were embedded in paraffin blocks and then cut into sections with a thickness of 4 μm. These sections were subsequently subjected to a standard protocol for Sirius red staining. To determine the extent of positive staining (indicated by red coloring) in digital images of the Sirius-red-stained liver sections, ImageJ 1.53p software was utilized for quantitative analysis.

### 4.10. ACTA2 Immunostaining

Rabbit polyclonal antibodies against ACTA2 (1:800 dilution) were obtained from Thermo Fisher Scientific (Rockford, IL, USA) for immunostaining of ACTA2 in the liver sections. The peroxidase-labeled streptavidin–biotin technique was employed for immunostaining. The liver sections were incubated with the primary antibodies for 30 min, followed by multiple rinses with phosphate-buffered saline. Subsequently, goat anti-rabbit secondary antibodies were applied to the sections and allowed to incubate at room temperature for 30 min. The sections were then treated with an avidin–biotin compound and subsequently exposed to a peroxidase substrate solution for 5 min to mask any endogenous peroxide activity. Visualization of the sections was achieved using a DAB kit, and counter-staining was performed with Mayer’s hematoxylin. The tissue expression of ACTA2 was evaluated by determining the percentage of positive area relative to the total area, utilizing ImageJ 1.53p software.

### 4.11. Survival Analysis

In this study, Kaplan–Meier survival plots were utilized to evaluate the effectiveness of the treatments. Specifically, we compared the survival function of rats in the TAA/STA9090 group with that of the TAA group. The Kaplan–Meier survival plots visually depict, at each time point, the percentage of animals that survive, enabling a comparison of survival functions between groups over time. 

### 4.12. Determination of Liver Function and Oxidative Stress Markers 

The activity of Serum ALT, AST, and ALP was measured using colorimetric assays with commercially available kits from Bio-diagnostic (Giza, Egypt). To assess the presence of reactive oxygen species (ROS) in liver tissues, we employed a methodology that was previously described [57]. We employed commercially available kits from Biodiagnostic to evaluate the levels of malondialdehyde (MDA). MDA reacts with thiobarbituric acid to form a fluorescent red derivative, which was quantified following the kit’s instructions. Additionally, the spectrophotometric analysis of reduced glutathione (GSH), superoxide dismutase (SOD), and catalase (CAT) was carried out using commercially available kits also obtained from Biodiagnostic, following the provided instructions.

### 4.13. qRT-PCR for the mRNA Expression of Collagen, Type I Alpha 1 (Col1a1), TGF-β1, and NFκB p65

The liver tissue samples were subjected to total RNA extraction using the RNeasy Mini kit from Qiagen, a company based in Germany. The extraction process followed the protocol provided by the manufacturer and was conducted in an environment free of RNase contamination. The concentration and purity of the isolated RNA were assessed using a NanoDrop 2000 spectrophotometer from Thermo Scientific, Waltham, MA, USA To generate complementary DNA (cDNA), 1 μg of high-quality RNA was reverse transcribed using a QuantiTect reverse transcription kit from Qiagen, Hilden, Germany. This kit employs quantiscript reverse transcriptase for the conversion process. PCR amplifications were carried out using a Rotor-Gene Q thermocycler manufactured by Qiagen in Hilden, Germany. The PCR reactions utilized the SYBR Green PCR Master Mix provided by Qiagen, CA, USA. Gene-specific PCR primers were designed using Primer Express 3.0 software developed by Applied Biosystems, Foster, CA, USA (refer to Table 2). To confirm the specificity of the primers for the intended genes, BLAST analysis on NCBI was conducted for each primer. The relative expression levels of the target genes were determined using the comparative cycle threshold (Ct) method (2^−ΔΔCT^). The Ct values were normalized to the expression of the GAPDH gene, which served as an internal control within the same sample. Data analysis was carried out using the Rotor-Gene Q Software 2.1 provided by Qiagen.

### 4.14. Determination of Hydroxyproline Hepatic Content

The concentration of hydroxyproline in the liver tissue homogenate was determined using spectrophotometry. For this purpose, kits obtained from Sigma-Aldrich were employed. These kits utilize the interaction between oxidized hydroxyproline and 4-(dimethyl amino) benzaldehyde (DMAB) to produce a colored product. The intensity of the color is directly proportional to the concentration of hydroxyproline in the sample.

### 4.15. Measurements of Hepatic HSP90 and HSP70, p65 Binding Activity, and Serum TNF-α

The concentrations of HSP90 and HSP70 were measured utilizing kits obtained from Abcam (Cambridge, UK) and Novus Biologicals (Littleton, CO, USA), respectively. To assess the activity of NFκb p65 binding, an Abcam kit (Cambridge, UK) was utilized. The kit included a 96-well plate with a specific double-stranded DNA sequence containing the NFκB p65 consensus binding site (5′-GGGACTTTCC-3′) immobilized on its surface. The nuclear extract, containing active NFκB p65, was incubated with the plate, enabling the protein to specifically bind to the oligonucleotide. NFκB p65 detection was performed using a primary antibody that specifically recognizes an epitope of NFκB p65, which is accessible only when the protein is activated and bound to its target DNA. Subsequently, a secondary antibody conjugated with HRP was applied, resulting in a colorimetric readout at an optical density of 450 nm. This provided a sensitive measurement of NFκB p65 binding activity. Prior to the experiment, nuclear extracts were prepared following the instructions provided by the nuclear extraction kit (Abcam), and the aliquots were stored at −80 °C after determining their protein concentration. Serum levels of TNF-α were determined by a kit that was obtained from CUSABIO (Wuhan, China).

### 4.16. Measurements of the Levels of TGF-β, MMP-9, TIMP-1, PDGF-BB, HIF-1α, and VEGF

TGF-β and tissue inhibitor of metalloproteinase- 1 (TIMP-1) levels were quantified using kits obtained from CUSABIO. The measurement of matrix metalloproteinase-9 (MMP-9) levels was performed using a kit from Elabscience Biotechnology Co., Ltd., located in Wuhan, China. For the determination of platelet-derived growth factor-BB (PDGF-BB) and hypoxia-inducible factor-1 alpha (HIF-1α) levels, we utilized kits provided by Abcam. Vascular endothelial growth factor (VEGF) was determined using a kit obtained from Invitrogen, based in Carlsbad, CA, USA. All procedures followed the manufacturers’ instructions. 

### 4.17. Measurement of the Proteasomal Activity in the Liver

The experimental groups consisted of the TAA group and the TAA/STA9090 group. Isolated livers (10 mg each) were homogenized with Dounce tissue homogenizer. Then, 100 μL of 20S IP assay buffer was added. It was homogenized on ice for 5 min to lyse the tissue. The lysate was centrifuged at 10,000× *g* and 4 °C for 10 min and the supernatant was collected for the assay. The volume of the sample was adjusted to 50 μL with 20S IP assay buffer, and the sample was added into two wells labeled as sample and inhibitor. For the inhibitor well (to isolate the proteasome activity from other protease activities in the samples), 1 μL of 20S IP Inhibitor was added and the volume was adjusted to reach 50 μL with 20S IP assay buffer. For the substrate background control well, 50 μL of 20S IP assay buffer was used. For the positive control well, 2 μL of 20S IP positive control was added and the volume was adjusted to 50 μL with 20S IP assay buffer. Fluorescence was recorded at excitation/emission wavelengths of 350 nm/440 nm at 37 °C, respectively. The zero standard reading was subtracted from all standard readings, and we plotted the AMC standard curve. Next, we subtracted the Inhibitor reading from all sample readings to obtain corrected sample readings. Subsequently, we applied these corrected sample readings to the previously generated AMC standard curve to quantify the amount of AMC present in the sample in pmol. One unit of 20S immunoproteasome is the amount of enzyme that produces 1.0 μmol of AMC per minute at 37 °C. The protein concentration of each sample was determined using the BCA protein assay kit and the obtained values normalized by the protein concentration, unit/μg of total proteins ± SEM (duplicate per assay), were normalized to the proteasome activity of the TAA rat group, which served as the control. The data are presented as a fold change of the control samples, with the control samples set to 1. Six independent assays/group were conducted, and each assay was performed in duplicate.

### 4.18. Measurement of TβRII, p62, and BECN1

TβRII levels were measured using a kit provided by Abbexa, located in Cambridge, UK. For the determination of p62 levels, we utilized a kit obtained from MyBioSource Inc., based in San Diego, CA, USA. BECN1 levels were determined using a kit from CUSABIO.

### 4.19. Statistical Analysis

A statistical analysis was performed using GraphPad Prism v. 9. For group comparisons, a one-way ANOVA followed by Tukey’s post hoc test was conducted. An unpaired *t*-test was used to compare the means of two independent groups. The data are presented as the mean ± standard deviation. To compare the scores, the Kruskal–Wallis test was employed, followed by Dunn’s post hoc test. A significance level of 0.05 was used, indicating that a *p*-value below this threshold was deemed statistically significant.

## 5. Conclusions

STA9090 demonstrated promising effects in vitro and in vivo. It increased the hepatocyte survival rate, decreased the leakage of LDH, and improved liver function in TAA-exposed rats. Notably, STA9090 exhibited pleotropic effects by targeting proteins involved in inflammation and constraining collagen production, resulting in improved microscopic features of the rat livers. Our findings suggest that STA9090-induced HSP90 inhibition leads to the degradation of TβRII, a fibrogenic client protein of HSP90, through the activation of the 20S proteasomal degradation system. Importantly, this degradation mechanism is autophagy–lysosomal pathway independent. Additionally, HSP90 inhibition by STA9090 was found to destabilize HIF-1α and promote its degradation, leading to reduced transcription of VEGF. Furthermore, STA9090’s ability to deactivate the NFκB signaling pathway highlights its potential as an anti-inflammatory and antifibrotic agent. By hindering NFκB signaling, STA9090 may mitigate the inflammatory response and prevent the development of fibrosis. While the induction of HSP70 expression following HSP90 inhibition shows promise, further studies are required to fully understand its role and therapeutic potential in preventing the development of liver fibrosis. In this study, we have shed light on the potential of STA9090 as a therapeutic agent for liver fibrosis. However, further research is needed to elucidate the underlying mechanisms and fully harness the therapeutic benefits of targeting HSP90 and related pathways. By advancing our knowledge and exploring innovative approaches, we can pave the way for improved management and treatment options for liver fibrosis.

## Figures and Tables

**Table 1:** Study design.

Exp. Groups	6 Weeks
Normal (*n* = 8)	-
STA9090 (*n* = 8)	STA9090 (20 mg/kg/every other day, i.p.)
TAA (*n* = 15)	TAA (150 mg/kg twice a week, i.p.)
TAA/STA9090 (*n* = 12)	TAA (150 mg/kg twice a week, i.p.) and STA9090 (20 mg/kg/every other day, i.p.)

i.p., intraperitoneal; TAA, thioacetamide.

**Table 2:** Primer sequences for qRT-PCR.

Gene	GenBank Accession	F	R	Amplicon Size (bp)
Col1a1	NM_053304.1	5′-GACATGTTCAGCTTTGTGGACCC-3′	5′-AGGGACCCTTAGGCCATTGTGTA-3′	120
TGF-β	NM_021578.2	5′-CTTCTCCACCAACTACTGCTTC-3′	5′-GGGTCCCAGGCAGAAGTT-3′	139
NFκB P65	NM_199267.2	5′-TTCCCTGAAGTGGAGCTAGGA-3′	5′-CATGTCGAGGAAGACACTGGA-3′	185
GAPDH	NM_017008.4	5′-TCAAGAAGGTGGTGAAGCAG-3′	5′-AGGTGGAAGAATGGGAGTTG-3′	111

## Data Availability

The data presented in this study are available from the corresponding author upon request.

## Abbreviations

ALT, alanine aminotransferase; AST, aspartate aminotransferase; CAT, catalase; Col1a1, collagen, type I alpha 1; DMEM, Dulbecco’s modified Eagle medium; ECM, extracellular matrix; EMT, epithelial–mesenchymal transition; HIF, hypoxia-inducible factor; HIF-1α, hypoxia-inducible factor-1 alpha; HSCs, hepatic stellate cells; HSP90, heat shock protein 90; LDH, lactate dehydrogenase; MDA, malondialdehyde; MMP-9, matrix metalloproteinase-9; NFκB, nuclear transcription factor kappa B; NII, necro-inflammation index; PDGF-BB, platelet-derived growth factor-BB; PHD, domain-containing proteins; pVHL, tumor suppressor von-Hippel–Lindau protein; ROS, reactive oxygen species; SOD, super oxide dismutase; TAA, thioacetamide; TGF-β1, transforming growth factor-β1; TIMP-1, tissue inhibitor of metalloproteinase- 1; TβRII, TGF-β receptor type-2; VEGF, vascular endothelia growth factor.
